# Some Assembly Required: Player Mental Models of Videogame Avatars

**DOI:** 10.3389/fpsyg.2021.701965

**Published:** 2021-07-15

**Authors:** Jaime Banks, Nicholas David Bowman

**Affiliations:** College of Media & Communication, Texas Tech University, Lubbock, TX, United States

**Keywords:** avatars, videogames, mental models, assemblage, semantic network analysis

## Abstract

In playing videogames, players often create avatars as extensions of agency into those spaces, where the player-avatar relationship (PAR) both shapes gameplay and is the product of gameplay experiences. Avatars are generally understood as singular bodies; however, we argue they are functional and phenomenological assemblages—networks of social and technological components that are internalized by players as networks of knowledge about the avatar. Different PARs are based on different internalizations (i.e., mental models) for what an avatar is and why it matters. Toward illuminating nuances in PARs, we examine the content and structure of players’ internalizations of avatars as evidenced by descriptions of those digital bodies. Secondary analysis of *N* = 1,201 avatar descriptions parceled them by PAR type (avatars as asocial Objects, psychologically merged extensions of Me, hybrid me/other Symbiotes, and authentically social Other). Aggregated descriptions for each PAR type were subjected to semantic network analysis to identify patterns in salient avatar components, and then qualitatively compared across the four PARs. Results indicate component clusters that are universal to PARs (demographics and body features), common to three of four PARs (time, appearance, clothing, and player agency), and idiosyncratic to specific PARs (significance, character narratives, game dynamics, liminality, and gratifications). Findings signal the importance of theoretically engaging avatars as assemblages both (a) influenced by player-avatar sociality and (b) that contribute (in part and whole) to antecedents, processes, and effects of gameplay.

## Introduction

In videogames, bonds between users and avatars are complex and multifaceted. Although much research has explored the psychological merging of player and avatar (i.e., identification), other evidence suggests that player-avatar relationships (PARs) span a range of sociality. This sociality is represented in four heuristic categories, in which players approach their avatars as (a) asocial “Object” orientations for challenge and competition (b) psychologically merged “Me” orientations for social play (c) hybrid “Symbiote” orientations toward identities and agency negotiations, or (d) authentically social “Other” orientations often marked by escapism (Banks, 1995). These categories are further informed by players’ assessments of relational closeness with, anthropomorphic autonomy of, sense of control over, and critical concern for specific avatars (Banks and Bowman, 2016a; Banks et al., 2019).

Although this research shifts attention toward a dyadic (rather than monadic) approach to PARs (Banks, 1995), a preponderance of scholarship still characterizes avatars as monolithic entities—singular bodies with singular personas engaged in a singular manner akin to users’ physical bodies (cf. Yee et al., 2008). Yet structurally and functionally, avatars are best understood as complex *assemblages* of discrete technological and anthropomorphizing components (Banks, 2018), and players’ experiences of avatars may be correspondingly understood through the lens of mental models—dynamic cognitive structures representing the internalization of external phenomena (Craik, 1943). To examine the ways in which gamers variably internalize avatars, the current study examines a corpus of players’ descriptions of favorite avatars to discover their form and content and to identify key similarities and differences across PAR types.

### Players, Avatars, and Social Relations

The term “avatar” is appropriated from Hindu scripture (de Wildt et al., 2019). It is rooted in Sanskrit and is commonly translated to “incarnation” in English, where avatars are the representation of gods manifest on Earth for specific tasks through a variety of forms (Mukherjee, 2012). A clear example of this can be found in the *Dashavatara*, which recounts the 10 incarnations (*swaroop*) of Vishnu. As the Hindu god of preservation, Vishnu comes to Earth in various avatars, each aimed at thwarting a specific danger to humanity. For example, as the Kurma (the second incarnation), Vishnu adopted a half-tortoise, half-human avatar who balanced Mount Mandara on his shell during the “Samudra Manthan”—the churning of the oceans. As the Parashurama (sixth incarnation), Vishnu took the guise of an ax-wielding priest (Brahmin) who fought against a warrior class (Kshatriyas) misusing their power to oppress others. As the Gautam Buddha (ninth incarnation), Vishnu established a peaceful path for humanity in the foundation of Buddhism and the establishment of the Noble Eightfold Paths. In these examples, we can understand avatars as tangible embodiments of ephemeral forces that take forms commiserate with a given task.

Applied to digital worlds in videogames, avatars have been characterized as users’ “casting off of the flesh” in shifting attention and intention away from physical worlds and toward digital worlds (a decarnation; Boellstorff, 2008).^1^ As players’ experiences in digital worlds are (to some extent) mediated by avatars, many approaches to study PARs presume that the former and the latter “psychologically merge” (Lewis et al., 2008, p. 515). Some suggest that PARs are “staggeringly simple: the player is the avatar and vice versa” (Mukherjee, 2012, para 8). This theorized merging relies, in part, on how players identify with (or as) their avatars, suggesting that when players step into their digital containers, they experience a “temporal shift in self-perception through adoption of valued properties [of another]” (Klimmt et al., 2009, p. 351). A polythetic model for player-avatar identification (Downs et al., 2019) contends that players can perceive variable levels of physical similarity, embodiment, value homophily, perspective-taking, and wishful identification with their on-screen avatar—none required but all contributing to felt identification.

However, this focus on monadic identification as the *de facto* way in which players engage their avatars does not permit for a broader consideration of the full range of possible PARs. For example, in many videogames players engage digital worlds through the diegetic lens of an established character—*another*, rather than themselves. Likewise, in digital experiences where players craft their own avatars, there is no requirement that avatars one-to-one represent players’ corporeal self. Recognizing degrees of self-differentiation (rather than identification) between players and avatars permit reconsideration of how these relationships can be *authentically social*—that is, players can variably perceive avatars to be separate agents and form a social bond with them.

Bowen (1978) suggests that a key distinction between social relationships and dependent connections is that one (here, the player) must see the other (the avatar) as a distinct entity with its own subjectivity. For instance, the player creates a mage avatar: In play, the player enters a command into the system instructing the avatar to cast a spell; then, the avatar communicates back to the player that it does “not have enough mana” (see Kudenov, 2018). Here, player and avatar are exchanging information based on the native communicative abilities of each (see Banks and de Graaf, 2020). The player encodes meaning by pushing a button, the message is conveyed through the game medium, and the avatar decodes the message according to its programming; in turn, the avatar encodes a message according to its programming, the message is conveyed through the game’s software to the game’s supporting hardware and peripherals (i.e., computer speakers), and the player decodes it by interpreting the words. In these ways, if one rejects anthropocentric accounts of what counts as communication or meaning-making, player-avatar relations are inherently *functionally* social in that each entity is encoding and decoding messages as it engages the other. As such, variations in PARs are less about the formal dynamics of a relation and more about the extent to which the relation is *perceived* as social.

### Player-Avatar Relationships

As argued here and in prior work (Banks, 1995; Banks and Bowman, 2015, 2016a,b; Banks et al., 2019), PARs can be broadly parceled as a function of sociality (from phenomenologically asocial to social) with four discrete relationship types forming along this continuum: avatar-as-Object, avatar-as-Me, avatar-as-Symbiote, and avatar-as-Other.

#### Avatar-as-Object

Anchored in the asocial, many players approach their on-screen with an Object orientation—seeing their avatar as a “toy,” “tool,” “puppet,” or “object” existing merely for purposes of gameplay (Banks and Bowman, 2015). When seen as objects, avatars are engaged as mere tools with which to play the game such that players feel little emotional investment in them and experience little recognition of their legitimacy as characters (Banks and Bowman, 2016a,b). Players engaging avatars as objects are mostly focused on challenge and competition gameplay (Banks, 1995; noting these to be prominent videogame motivations, see Sherry et al., 2006; Yee, 2006), and likewise, enjoyment is mostly derived from players’ abilities to succeed through their avatars (Klimmt et al., 2009; Oliver et al., 2016). Koles and Nagy (2016) found evidence that some gamers viewed their avatars as collectible objects, serving as indicators of status and personal accomplishment.

#### Avatar-as-Me

For some players, avatars are extensions of the players themselves—very much associated with notions of avatars as an agentic “extensions” or self “representations” of players (Banks and Bowman, 2015). Banks (2015) explained that these players tend to engage digital worlds in more or less the same ways that they engage physical worlds, as the on-screen avatar is carefully crafted to *literally* represent the player, although this representation can take several forms. With respect to sociality, these “Me” orientations cannot be construed as social relationships^2^ because they largely represent a monadic or merged orientation: The player and the avatar are one and the same entity (perhaps the most literal application of the notion of an avatar)—Me orientations do not distinguish between themselves and the avatar (Ko and Park, 2020). Me orientations can be understood through various theories of the self, including social identity theory (Teng, 2017) and self-affirmation theory by Teng (2019).

#### Avatar-as-Symbiote

Although Object and Me orientations appear to dominate most study samples (see Bowman and Banks, 2021), some gamers have meaningful relations with avatars in a blended sense, seeing some of themselves in avatars but also seeing elements of a unique and authentic social other (Banks, 1995, 2013). These relations may be described as symbiotic—the avatar manifests an entanglement of self and other. Players who are working through interpersonal conflict—such as those questioning their gender identity, coping with disability, or working through aversive (potentially identity-threatening) experiences—are inclined to engage avatars as a sort of identity laboratories (Banks, 2013; cf. Nakamura, 1995; Turkle, 1995). Such Symbiote relations break from Me relations in that they ascribe more agency and personality to avatars as demi-persons, but still see them as anchored in the self. For example, whereas a Me-relation player might create an avatar that represents an idealized version of the self, a Symbiote-relation player would experience an avatar as a separate social entity that serves as an affective and behavioral exemplar (i.e., modeling possible selves; Markus and Nurius, 1986). Clark et al. (2018) found evidence of such hybrid identities in avatars created for exergames, in which players discussed a tension between presenting current and ideal body types (although others framed their avatar more as an Object to engaging exercise sans any further connection). In some cases, the Symbiote relationship can represent a stage of identity transference in which players begin to see themselves in their avatars or vice versa [Koles and Nagy, 2021; also suggested in Banks (2013)].

#### Avatar-as-Other

At the far end of the player-avatar sociality spectrum, there are gamers who label their avatar using terms (such as “partner” or “person;” Banks and Bowman, 2015) suggesting that avatars are authentic and self-differentiated social entities—not so different phenomenologically from friends. Although these relations are not limited to role players, Other orientations do tend to rely on headcanon (see McKnight, 2018)—an original narrative that aligns with the broader world narrative, situating it diegetically as having life histories, relationships, experiences, and goals. Such orientations are common in role-playing games, for example, where gamers are often tasked with helping another negotiation through a given quest or series of tribulations that are wholly contextualized within the gameworld. As these relations rely on engagement with canonical or original narratives, Other PARs are characterized by socioemotional motivations and needs (Banks, 1995) and so often engender feelings of relatedness with in-game characters (see Oliver et al., 2016). An autoethnographic account by de Wildt et al. (2019) provides several examples of the interpersonal intimacy common for players engaging their avatars as authentic social Others. Burgess and Jones (2018) found evidence of players feeling that their avatar “broke character” during gameplay—that the avatar had a distinct personality from the player, but often times were forced to act “out of character” by either the player’s or the game designers’ hands.

### Avatars Are Assemblages in Practice and Perception

In considering how players experience these varied bonds with avatars, it is prudent to take a step back and reconsider the substance and functioning of what players are actually bonding with. We have defined an avatar as a body that extends agency and (sometimes) identity into a gamespace. Importantly, though, in the same way that human bodies are complex networks of tissues, structures, processes, and energy, so too are avatars complex assemblages of social and technological components (Steinkuehler, 2008; Giddings, 2009; Taylor, 2009; Banks, 2018). Put another way, an avatar is a coming together of elements existing in complex relations, convened by some agent (here, the player; Nail, 2017). For instance, their technological anatomy may include behavioral scripts, skill statistics, polygonal structures, and even glitches, while their anthropomorphizing (i.e., social) anatomy includes embodied features, modes of gesturing and movement, moral alliances, and character relationships. As these components constellate in the course of gameplay, the potential for players to connect with avatars emerges at the intersection of the parts and the whole (Banks, 2018).

Put more directly, avatars are not only operational assemblages but also *phenomenological assemblages*. That is, people experience avatars to some extent as collections of discrete elements such that players involved in different PARs may actually be having different relationships with different assemblages—and the nature of those assemblage relations may explain the gameplay motivations and gratifications known to emerge from different PARs.

Regarding technical and ludic components, evidence indicates that some players focus intensely on avatar statistics as they seek to empower an avatar (Ask, 2017) while others emphasize mechanics mastery toward achievements and the building of cultural capital through avatars (Korkeila and Hamari, 2020). Some work toward manipulating avatars to subvert normative gameplay (de Peuter, 2015) while others still exploit glitches to explore forbidden gameplaces or novel forms of play (Johnson, 2018). For anthropomorphizing social components, evidence indicates players have attachments to specific body parts like hair and feet (Banks, 2017) and may carefully attend to markers of gender-, race-, and sexuality-group identities (Martey and Consalvo, 2011). Sometimes personality and embodied components are more holistic or heuristic as when avatar characters are narratively framed as “good” or “evil” (Melenson, 2011) while others are more piecemeal as players assemble multimodal, symbolic representations of a character from tokens and visuals (Banks et al., 2018). Importantly, some technological components have patterned connections with other anthropomorphizing components, as when game functions and aesthetics influence avatar names (Hagström, 2008) or dictate that certain gameplay roles require avatars to wear certain types of clothing (e.g., healers often wear light cloth gear).

In addition to the assembled nature of avatars, it must be acknowledged that the avatar and player are, together, an assemblage (a cyborg of sorts; see Zylinska, 2002) and are situated within and across assembled spaces (i.e., the liminal gameplay environment). Because the boundaries of assemblages are often difficult to demarcate (see Latour, 2005), the bond with an avatar-as-assemblage—as it is subjectively experienced—may also incorporate components of the player and the environment, such as motivations and gratifications (e.g., Yee, 2006), gameworld and interface elements (e.g., Taylor, 2009), and gaming-culture norms and practices (e.g., Consalvo, 2007). These broad potentials for the constitution of avatars as phenomenal assemblages warrant careful inquiry into what, exactly, matters to players as the engage and understand avatars—in whole or in part—in and around games.

### PARs as a Function of Mental Models

The subjective experience of avatar-assemblages is perhaps best understood through the lens of mental models (MMs). MMs are cognitive frameworks that represent a person’s internalized knowledge about some external thing, consisting of knowledge “tokens” or quasi-pictorial representations; each token represents some discernible or abstract component of that thing, where the MM structure reflects one’s understanding of the actual or possible structure of that thing (Johnson-Laird, 1989, 1995; see Rickheit and Sichelschmidt, 1999 for a review). The elements composing MMs are drawn from direct and indirect experiences (Seel and Strittmatter, 1989) such that MMs can be understood as a way of knowing a thing (such as an avatar) and that knowing guides how people approach new experiences (Craik, 1943) and think about possibilities (Johnson-Laird and Byrne, 2002).

If (as argued) avatars are functional and subjective assemblages and MMs are internalized knowledge structures representing a thing, then understanding player-avatar relations require attention to how players hold MMs for their avatars. By understanding how players internalize the avatar-assemblage, we may better understand how those internalizations contribute to and vary across PARs. Importantly, because MM content is causally linked to people’s attitudes and intentions toward some social technologies (Banks, 2020), understanding this internalization may be key to understanding how PARs influence subjective experiences of play. Thus, we ask (RQ1) what is the content and structure of players’ mental models for avatars in the four primary PARs?

## Materials and Methods

To address the posed research question, we conducted a secondary analysis of players’ open-ended descriptions of their avatars from existing datasets in which participants were asked to both (a) describe their in-game avatars and (b) indicate which one of the four heuristic PAR types (Object, Me, Symbiote, and Other) best described their connection with this focal avatar. These open-ended responses were subjected to semantic network analysis to identify clusters of semantically and structurally related words as representative of assembled MM knowledge tokens.

### Participants

Data were aggregated from *N* = 1,201 respondents from past research investigating PARs, including studies on (1) changes in character appearance (*n* = 482; World of Warcraft; Banks, 2017) (2) sense of place (*n* = 370, Fallout 76; Bowman et al., 2020) (3) memorable experiences with avatars (*n* = 309; various; unpublished data, see online supplements), and (4) military gamers (*n* = 52; various; Banks and Cole, 2016)—referred to as datasets 1–4 (DS1-4) Among these descriptions, most referred to World of Warcraft avatars (*n* = 591) and Fallout 76 (*n* = 367) as a function of DS1 and DS2’s emphasis on those games as well as prevalence within the other datasets; these were followed by Guild Wars (*n* = 37), EVE Online (*n* = 20), Skyrim (*n* = 12), and other games (*k* = 96 other game titles named in *n* = 174 remaining player narratives; see OSF for complete list of games mentioned). Across all datasets in our secondary analysis, participants were *M* = 28.17 years old (*SD* = 8.67, range 18–74, median 26) and 73.7% male, 23.7% female, 1.2% nonbinary, and 1.4% not reporting. All data and analysis scripts for this project are shared freely *via* an Open Science Framework project folder at https://osf.io/8n9mp/.

### Measures

#### Open-Ended Avatar Description

Because MMs are unobservable internalizations, they must be externalized—often most readily accomplished through narration (see Rickheit and Sichelschmidt, 1999). To elicit the internalization of players’ avatar, the prior studies’ participants were asked to describe an avatar, with slight language variations reflecting the individual studies themselves. DS1 is based on a request for participants to name a favorite avatar within the game and then to “describe that avatar.” DS2 data resulted from eliciting a most-played avatar’s name and then asked players to “describe [name]‘s appearance.” DS3 includes responses to requests to name their favorite avatar and then “offer a brief description of that avatar, in your own words.” DS4 data resulted from requesting the name of a videogame that was important to the player, to then name a favorite avatar in that game, and then to “describe this avatar.” As these datasets were drawn from discrete studies, the variation in language is acknowledged as a limitation of the current secondary analysis (in particular, DS2’s attention to appearance over more general descriptions); however, all prompts are similar mental-model elicitations in that they work to externalize players’ internalizations of their avatars.

#### Player-Avatar Relationship Type

All four datasets used a single categorical item to capture the heuristic PAR type for the specific, named avatar. Players indicated that “This avatar is merely an object on a screen” (Object; *n* = 400), “This avatar is me” (Me; *n* = 277), “This avatar and I are part of each other” (Symbiote; *n* = 329), or “This avatar is a separate being” (Other; *n* = 195).

### Analytical Approach

In externalizing the avatar MM by responding to the elicitation, the description is engaged as a tool to infer the content of the MM. The description’s words represent the content of the MM (i.e., knowledge tokens) and the structure (e.g., grammar and word co-location) represents the relations among the content elements; analyzing the semantic structure of a text allows for inferencing of the MM components that are accessible to a person as they narrate their understanding of the avatar (see Sowa, 1992). As argued by Banks (2020), examining content of MMs within and across individuals is a challenging affair because of the great variation among individual MMs—so much so that it is unlikely that there would be a “canonical form” by which cases could be compared (Woods, 1975, p. 16). This challenge is addressed by aggregating texts according to a feature of interest (here, PAR types) and constructing a semantic-network model for that corpus. In other words, we address the question of PAR-specific mental models by examining avatar descriptions aggregated by PAR-type, such that the unit of analysis is the relationship type rather than any one instance of it.

Prior to analysis (and to facilitate data sharing), elicited avatar descriptions were anonymized (replacing avatar name- and guild-mentions with NAME and GUILD) and were standardized for variations in language (e.g., MMO instead of MMORPG; see online supplements for all standardizations). Data were vetted to ensure valid descriptions of avatars versus an irrelevant response to the prompt (e.g., “I do not know”) and matching of an avatar to the game (e.g., Mario does not appear in *Guitar Hero*). Data were then prepared for the semantic network analysis by removing all terminating punctuation (periods, exclamation points, and question marks) from within the response and ensuring a period at the end of the response such that the analysis software (Leximancer; Smith and Humphreys, 2006) would recognize the whole response as a single unit of analysis. Obvious misspellings were corrected to ensure they were similarly accounted for in analysis (e.g., charismatic to charismatic, mohawk to mohawk).

**Figure 1:**
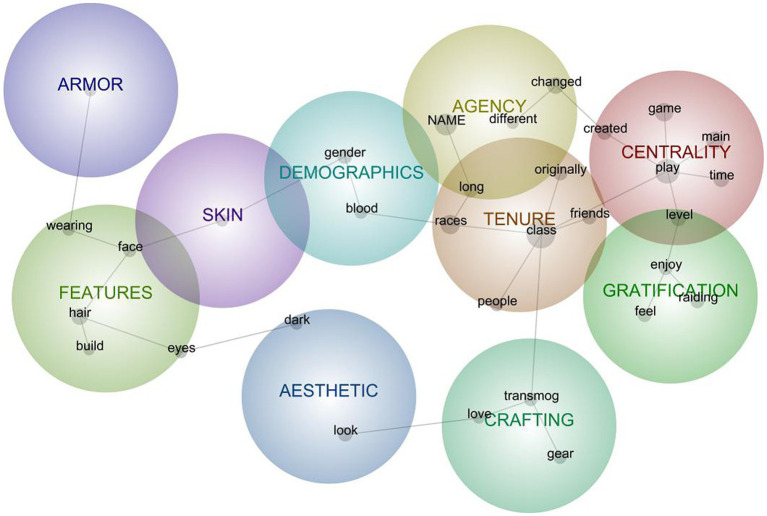
Semantic network model for aggregated descriptions of avatars in Object relations.

**Figure 2:**
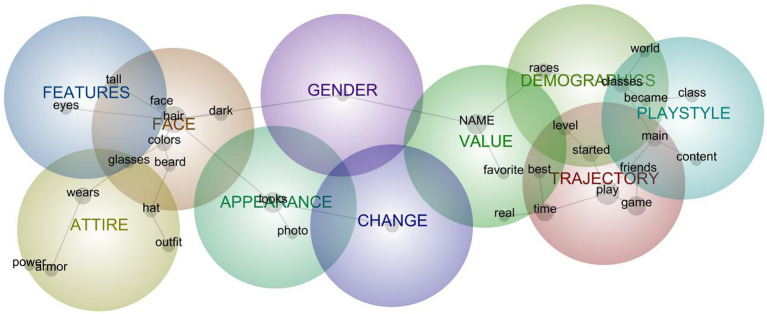
Semantic network model for aggregated descriptions of avatars in Me relations.

**Figure 3:**
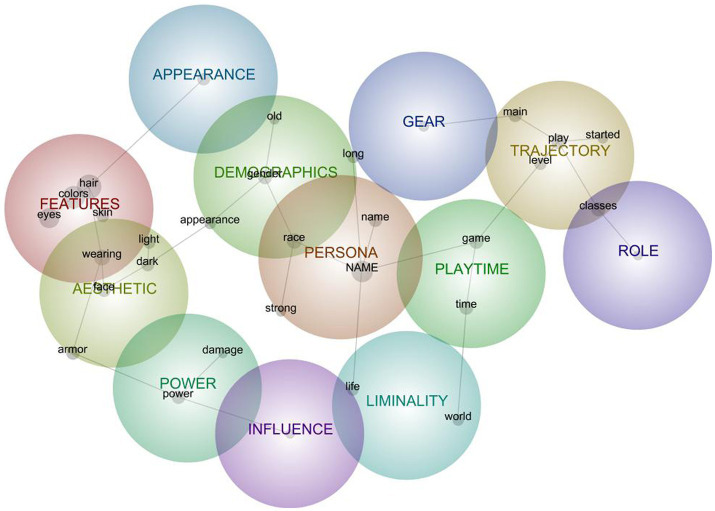
Semantic network model for aggregated descriptions of avatars in Symbiote relations.

**Figure 4:**
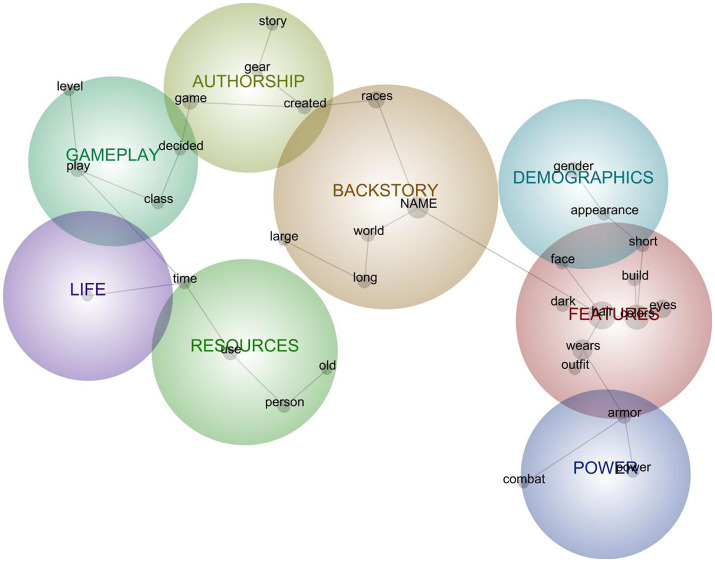
Semantic network model for aggregated descriptions of avatars in Other relations.

Avatar descriptions were parceled into four corpuses based on player’s self-reported PAR type, resulting in *n* = 400 Object, *n* = 277 Me, *n* = 329 Symbiote, and *n* = 195 Other type-aligned descriptions. Each corpus was independently subjected to semantic network analysis per Leximancer’s standard procedure: text processing (one sentence per block, merging word variants, and inclusion of name-like concepts), generation of concept seeds (removing words artificially injected by the prompt [e.g., “avatar”] and merging word classes [e.g., “brown” and “blue” as the object “color”]; see online supplements), generation of thesaurus, and generation of the concept map (i.e., semantic network map) based on the induced thesaurus (see OSF space for technical details of map generation; Leximancer, 2021 for analysis process details). This process resulted in four concept maps (one for each of the four PAR types) and accompanying catalog of data excerpts corresponding with each map’s concept-clusters. The maps (presented in Figures 1–4) were interpreted with consideration for heat mapping (clusters toward the red end of the color spectrum include concepts with greater gravity in the overall aggregate model); this evaluation was synthesized with an interpretation of the ranked list of words comprising each concept and of the specific data excerpts from which the concept-clusters were derived (see online supplements for complete outputs.) This interpretive procedure resulted in the naming of concept-clusters and thick descriptions of their constituent data. Throughout, the counts provided refer to the number of “hits” (i.e., word instances) in the source data; they are offered for descriptive purposes, as indicators of relative prevalence within each PAR-specific map.

## Results

### Aggregate Model for Avatars in Object Relations

The Object model comprised 10 clusters (Figure 1) interpreted as generally reflective of the following mental-model components (with cluster-comprising keywords in italics):

#### Centrality (*n* = 203)

The extent to which the avatar is the *main* vehicle by which they *play* the *game*, having spent the most *time* with, often *created* for a particular play purpose and often having the most advanced *level*. Sometimes, this centrality is a sort of specialness and sometimes, it is more utilitarian given the amount of time required to advance to higher levels: “It is the very first avatar that I have *created* and I have been *playing* him for as long as I have been *playing* [the *game*] I do not *play* any other avatars other than my *main* avatar.”

#### Tenure (*n* = 196)

Having used the avatar for a *long* time, often as part of dedication to a *class* or alignment with a certain group of *people* (especially *friends*); often the avatar was the one *originally* created when starting to play the game (versus having been adopted later), as when “It’s been my main for as *long* as I played [the game] … She is a hunter it’s been my favorite *class* since then.”

#### Agency (*n* = 130)

The history or ability to *change* the avatar (especially into a *different* expressed race, sex, clothing, or *name*) or taking up a resistance to *change* due to being well known or attached to those characteristics. Notably, descriptions suggest that despite sometimes frequent and dramatic changes that avatar is usually still characterized as the same avatar. For instance, one player noted: “I do like to *change* her hair and face tattoo occasionally though I usually keep it black or white.”

#### Features (*n* = 110)

Specific descriptions associated with hair, face, eyes, body type, or clothing, as with “Blonde *hair*, blue *eyes*, and medium *build*.” Although usually rote descriptions, they sometimes included motivations for avatars’ design, such as “She is modeled after me” or combinations of descriptions with backstories, as one having “a slightly chubby *face* from a comfy few years in the vault.”

#### Gratification (*n* = 75)

Entertainment characterized as *enjoyment* or *feelings* derived from gameplay experiences, most often connected to *raiding* experiences. Sometimes feelings were general or holistic (as in “the one I *feel* most comfortable playing” as a matter of class mechanics and experience) and sometimes more specific (e.g., “I *enjoy* the *feeling* that I am a holy warrior that protects the innocent from evil”).

#### Crafting (*n* = 58)

The making of an avatar’s appearance, through *transmogrification* (the WoW-specific practice of assigning an appearance to avatar *gear* while retaining its gameplay statistics), especially in ways that reflect aesthetics that players *love*. Although usually this crafting of gear appearance is described as a player activity (“I enjoy to find cool and unique transmog gear that makes me stand out…”) it is sometimes attributed to avatars’ preferences (“She likes to transmog mostly in rogue gear…”).

#### Demographics (*n* = 57)

Membership in diegetic social groups—those that are specific to the gameworld, as with *gender* and race (specifically *blood* elf) or class specialization (i.e., WoW-common *blood*-named specs or abilities). These were most often rote descriptions of character features.

#### Aesthetic (*n* = 50)

Overall avatar *looks*, especially in terms of *dark* hair or *dark* aesthetics. For instance: “*Visually* my goal has been to combine the *dark* aspects of the warlock lore like spikes and chains and skulls with a more mystical look—candles, books, pointy hats, glowy runes, etc.”

#### Armor (*n* = 38)

General references to gear, usually in its functional sense (“Dude in X-01 modified armor and legendary weapons.”) but sometimes references to aesthetic sets (“In hulking classic DK armor…”).

#### Skin (*n* = 21)

General references to an avatar’s exhibited bodily color (“lavender *skin*”) or tone (“with light *skin*”) or attributes (“she is rotten and her *skin* is slowly decaying…”).

Looking more closely at the relationships among these clusters, those most prevalent are related to tenure, centrality, agency, and gratification (i.e., together focusing on playing for long, playing well, and playing enjoyably), and they are all linked together, suggesting some degree of co-occurrence in player descriptions. The other six clusters represent less prevalent and more peripheral MM components, indicating that aesthetics or embodied features are secondary to gameplay in Object MMs. Even crafting (an otherwise creative endeavor) is linked with tenure through a connection to avatar “class” which suggested that even creative aesthetic authorship could be done in support of the diegetic combat role (see object attachments; Koles and Nagy, 2016, 2021).

### Aggregate Model for Avatars in Me Relations

The Me model comprised 10 clusters (Figure 2) interpreted to depict the following mental-model components (with induced concept keywords in italics):

#### Trajectory (*n* = 114)

Narratives recounting avatars’ persistence or evolution over *time*, most often conveying *play* activities (especially *starting* new ones or those having been important from the *start* of the player-avatar relation), the social importance to playing with *friends*, and the avatar’s status as a *main* character for playing. In other words, this cluster represents an internalization of the avatar’s origins and arc over a gameplay history—often in ways that entangled the avatar’s arc with the player’s arc. For instance, one player recounted that “He’s been my *main* for several years … He was *starting* to do heroic but my job got crazy and I do not have the hours to put into raiding.” Sometimes both avatar and player trajectories were also entangled with those of technologies, such as when, at the *time* EVE Online *started*, avatars were merely “kind of ugly” images and names, but one could more fully customize an avatar by shaping their skills.

#### Face (*n* = 84)

Features of the *face* and head, inclusive of *hair*, *beard, glasses*, and specific *colors* and tones thereof. Excerpts linked to this cluster were often descriptions comprising listed features, as with “well-kept *brown* hair and a respectably short *beard*” and “Asian female with *dark* skin, tattoos, *face* painted to look like a skull.”

#### Demographics (*n* = 65)

Membership in diegetic social groups—those *race*, *class*, and *level* features that position avatars in relation to others in the game*world*. These features were often presented in list format (“Retribution paladin blood elf. High level. Fun to play.”) but were sometimes also presented as situating the character in the game’s narrative *world*, as with a monk who “wishes to rid the *world* of evil” or a young upper-class man “thrust into a *world* he never knew existed.”

#### Appearance (*n* = 58)

Descriptions of the overall *look* of avatars (“tough *looking*”), similarity to self (“*looks* like me just dirtier”), or appreciation for the appearance (“*looks* awesome”). Of note, this clustering may be an artifact of the FO76 players having uploaded multiple images over time, so references to *photograph* were often suggestive of the “badge photo” were a specific kind of uploaded image.

#### Attire (*n* = 56)

The collection of clothing *worn* by the character, presented as the current or typical outfit and most often noting armor or hats. For instance: “Currently *wearing* the Firebreathers *outfit* with the Beer *Hat* Bottle Cap Glasses.” As with the appearance cluster above, this cluster is heavily influenced by FO76 respondents in that they narrated highly customizable outfits and often juxtaposed them with more utilitarian *power armor* inherent to that game.

#### Value (*n* = 53)

Articulations of valuing avatars according to representative or gameplay significance, manifesting in two forms. The first is affect or attachment to the avatar or its features as a *favorite* among other possibilities, as with “one of my favorite gaming avatars I’ve ever inhabited it grew to embody me and my playing style.” The second is *real*ness akin to legitimacy associated with *real* life (“a representation of my real life persona”) or *real* value in gameplay (“this has been my only real avatar”).

#### Playstyle (*n* = 33)

Particular affinity for, skill in, or utility of avatars’ class as a matter of play style or activities. Often the playstyle relevance of the avatar was a matter of *becoming*—or becoming accustomed to or skilled in—as when an avatar was created ironically but “I grew a liking to as I got better gear and became better at the class.” This cluster sometimes included references to the avatar’s relevance with respect to a specific kind of *content*: solo, PvE, old, and new.

#### Features (*n* = 24)

Specific descriptions of avatar body characteristics, specifically constellated around eyes and height, as with “Brown hair, blue *eyes*, white, scruffy, *tall*, medium build.”

#### Change (*n* = 19)

Extent to which the avatar has (not) changed over time. Some are artifacts of the FO76 longitudinal data collection (e.g., “Appearance has not changed.”), while others are articulations that “I could not ever delete or change her” or that some characteristics are periodically changed (“long hair that changes color every few weeks”).

#### Gender (*n* = 15)

Indication of avatar sex (usually a binary male or female) that was tied to an expression of gender identity. Generally, these are embedded in descriptions with other demographics, as with “A *female* Twi’lek” or “Depending on my mood [name] was a gender and androgynous, fully *male*, or fully *female* with a chest.” Although keywords refer here to sex, keywords were embedded in more general constructions of avatar gender.

Looking more closely at the relationship among these clusters, two groupings emerge somewhat opposite each other: aesthetics and gameplay. The aesthetics grouping includes detailed and tightly networked facial features, flanked by clusters representing features, attire, gender, and appearance. The gameplay grouping is nearly mirrors that structure with a complex core cluster representing play trajectory that is flanked by playstyle and diegetic demographics. That these two groupings are linked by the value cluster suggests the avatar-as-Me’s status as a favorite and its sense of realness could be core to that PAR, reflecting Me relations’ tendencies to foster identification through both liking and self-reflection (Bowman et al., in press).

### Aggregate Model for Avatars in Symbiote Relations

The Symbiote model comprised 12 clusters (Figure 3) interpreted as representing the following mental-model components (with induced concept keywords in italics):

#### Persona (*n* = 132)

The persona manifested by/in the avatar by design or emergence, with particular references to *names*, *race*, and the characteristic of *strength*. *Names* were drawn from famous authors, mathematical concepts, time periods, popular media characters, lore, or those used by players across games. In addition to descriptive references to *race*, avatar *race* was unpacked as a key personality feature, as when “Charr are a beast *race* in the game–militaristic as well” or how a race/class combination does not “seem particularly inclined toward shadow in the lore.” *Strength* was invoked both as a descriptor for physiques (“very strong human male”) as well as for player and avatar personality (the avatar *race* “most resembles how I see myself … I’m *strong*.”)

#### Features (*n* = 121)

Specific descriptions of avatar body characteristics, specifically constellated around *hair*, *colors*, *eyes*, outfits *worn*, and *skin*. Some were straightforward (“she has shortish red *hair*, glasses, and fairly pale *skin*”) while others gave commentary or background about specific features (“She exclusively wears heavy armor and weapons to emphasize her physical strength.”).

#### Trajectory (*n* = 117)

Recounting avatars’ persistence or evolution, most often conveying *play* activities—especially those that recount how the avatar or player *started*, the process of *leveling*, or how the avatar functioned as a *main*. Some stories situated the avatar within a narrative and/or franchise timeline: The avatar “was a bounty *hunter* that fought for the Rebellion in Star Wars Galaxies. I *played* Star Wars Galaxies from just after the New Game Enhancement patch August 2005 and several years afterwards.” Other trajectories included how the avatar came to become the player’s *main* avatar, sometimes from the outset of gameplay (“My *main* from when I *started* to today”) and sometimes according to shifts in game content or social groups (“New main, rerolled holy paladin”). Avatar *level* was used as a benchmark for marking particular events, as with “eventually got to *level* 66 before switching servers.”

#### Demographics (*n* = 79)

Membership in nondiegetic social groups—those that do not necessarily belong to the gameworld, as with *gender* and age—often reflected in the avatar’s *appearance*. Specifically, avatars were often identified as *male* or *female* were described as being crafted in line with specific notions of masculinity and femininity, and often characterized as *old*—reflective of the character, player, or account age.

#### Time (*n* = 77)

How much *time* is spent in the *game* with the avatar, and how that *time* is spent. Generally, this included descriptions of how the avatar was engaged in gameplay (“spent most of his *time* either raiding Molten Core with groups or hitting the nascent PvP battlegrounds”), but also included indications of time as an investment (“increasing my avatar’s power which would occupy all of my *time* in the *game* world”). In some cases, players referenced specific time periods (“There was a time when I would pull weekenders…”) during which the avatar was specifically played.

#### Aesthetic (*n* = 74)

Overall avatar visual impression, especially in terms of *light* and *dark* impressions as manifested in *armor* and *face*. Armor was often describes as achieving a specific kind of style, as when a player assigned clothing such that it would “have a lot of attitude and be cool so he had a very serious but also expressive and edgy style to him.” *Light*ness referred to the brightness or goodness of the aesthetic (e.g., adhering to the “dark or light side of the force”), to the depth or intensity of the aesthetic or feature (“a light scar”), or to the weight of the armor (“mostly wears light armors”). *Dark*ness generally referred to intensity, as with “*dark* red *face* tattoos” or “heavy *dark* makeup.” References to face most often illustrated that part’s contribution to the aesthetic, as evidence of a personality (“her *face* is scarred from battle damage and she’s grubby”) or aesthetic (“the left side being much *brighter* and the right side of the *face* much *darker*”).

#### Liminality (*n* = 49)

Situatedness of avatars within/between *worlds* and as having/between *lives*. Players frequently depicted the avatar as having a particular narrative and history both within and across game and non-game *worlds* (“has family history that dates back 250 years in game *world* and about 15 years in the real *world*”), and as being in tension with their existence in the *world* of everyday *life* (“he represented a version of me that could get away with things I could not in the *real* world.”). In tandem, the avatar was often positioned in relation to “real *life*.” Sometimes there is a crossing of the diegetic boundary (“He exists mainly as a virtual entity but his spirit has also planted roots in real *life*”) and sometimes is situated squarely within world narratives but contrasted with one’s own life (“Fearless always willing to go into battles for a cause … Fighting for the top with no restrictions. The avatar that almost ruined my *life*.”)

#### Power (*n* = 44)

References to strength and force in terms of abilities (“abilities circle around channeling her rage into a *power* that effectively lets her withstand more *damage*”), narrative framings of those abilities (“wreaks havoc with a giant mace and the *power* of the Light”) specific kinds of gear (“currently in *power* armor”), and personality (“faces life with unshakable faith and an unwavering power of will”).

#### Appearance (*n* = 40)

Descriptions of the overall *look* of avatars (“I usually transmogrify his gear so he *looks* a bit like Indiana Jones.”), similarity to self (“try to make my avatar *look* somewhat like myself but within the game parameters”), or appreciation for the appearance (“it just *looks* badass”).

#### Gear (*n* = 19)

Avatar equipment (clothing and weapons) garnered as a matter of achievement (“my only focus for 8 + months in terms of gearing and raiding”) or visual appeal (“I usually transmogrify his gear so he looks a bit like Indiana Jones”).

#### Role (*n* = 17)

Functional or social purpose within a *guild*. This role is sometimes grounded in the avatar’s persona or gameplay value (going from “a lowly nameless individual to one of the biggest guild’s leader”) or in that of the player (“I have been my *guild’s* main tank for the previous 18 months”).

#### Influence (*n* = 11)

Impacts on *people*. *People* here most often referred to in-game characters (“a Hero of the Wasteland restoring hope and civilization to ordinary people”), but sometimes ambiguously to other players or players and characters jointly (“a beam of sunshine until it’s time to shoot people then she’s silent and efficient.”)

The Symbiote model featured relatively low density, with its clusters rather simple in structure (most with only a few keywords) and few connections among them. This is perhaps unsurprising given the inherent flexibility and variability with Symbiote relations, where the source corpus of avatar descriptions likely included of relations with varied player/avatar entanglements. That the persona cluster was as (a) the most prevalent and (b) the most central to the model reflects Symbiote relations’ tendencies to characterize avatars as simultaneously “me” but “not-me.” In particular, that persona-cluster tokens were connected with liminality is particularly reflective of this PAR’s characteristic engagement of avatars to solve identity challenges (Banks, 1995) at the intersection of embodied identities (clusters toward the left of the graph, inclusive of power) and agentic identities (play-descriptive clusters toward the right).

### Aggregate Model for Avatars in Object Relations

The Other model comprised eight clusters (Figure 4) interpreted to indicate the following mental-model components (with induced concept keywords in italics):

#### Features (*n* = 99)

Specific descriptions of avatar body characteristics, specifically constellated around *hair*, *colors*, *eyes*, *outfits worn*, *armor*, height, *build*, *face*, and *dark*ness of these features. Descriptions of *hair* (of various *colors*, lengths, and textures) were central to this cluster, with relatively detailed descriptions often attributed to avatars’ own tendencies or preferences, as when an avatar “*wears* a *dark red* leather coat with a tail that extends half way down her thighs over a thin *red* and *gray* chainmail vest.”

#### Backstory (*n* = 98)

Formal role play narratives or more latent headcanon that marry descriptors (*race* and appearance) with the character’s situation in the game *world*. Often beginning with “[*name*] is …,” these narratives position avatars in relation to a home *world*, as having a role in or disposition toward the *world*, and as having traveled the *world* in the course of its (often *long*) life. Sometimes these were sage-like dispositions, while others were more naïve (e.g., “Her thoughts and perception of the *world* is innocent and very childlike.”). Backstories often combined *racial* lore from the game universe with player-created narratives, as with an avatar canonically described as “a *human* ranger who was born in Ascalon and had to escape the City of Ascalon after the Searing” along with its emergent gameplay history, “He is accompanied by his pet … They have both traveled the *world* and saved it multiple times from Gods, evil characters, or dragons.”

#### Resources (*n* = 66)

Assets engaged in gameplay activities, on two levels: Diegetic assets *used* by avatars (force, armor, weapons, magic, mounts, and knowledge)—generally or in *time*-specific events—that, in turn, manifested the avatar as a resource *used* by players (name, caricature, spec, gestures, and *persona*), especially as a vehicle for specific kinds of play. Resources as engaged by the avatar exemplify the kind of *person* the avatar is seen to be—e.g., kind, free, terrible, knowledgeable, fictional, normal, quirky, and professional.

#### Authorship (*n* = 61)

Phenomenal composition of avatars within the *game* space, as a thing *created* at the intersection of *story* and *gear*. This *creation* is situated in relation to the *game* (as players go “back into,” “within,” or work “outside of” the *game*) or the *game* content is a resource for *creation*. The avatar’s *gear* is something “gotten,” “loved,” or “achieved.” *Creation* work relies in part on stories that are “crafted,” “invested in,” “allowed,” or “acquired” over time. Authorship constellates gameplay, narrative, and item creation, as when an avatar was described as “wearing a white headdress shoulder pad and cloak created from the very first polar bear she slayed in the cold lands of Winter spring the gloves are created from the Moon stags and the white wooly boots crafted with the fur from the Mammoths in Northrend.”

#### Gameplay (*n* = 53)

Activities and dynamics of *play*, inclusive of avatar *class* (and associated playstyle), *level* (and activities engaged into achieve that level), and the *decisions* made by players in the course of those activities. Notably, these descriptions leaned toward characterizing *levels* less as markers of progress or achievement and more as processual or experiential phenomena as in “the *leveling* process,” and *classes* less as markers of function and more in terms of enjoyment as in “I quickly fell in love with the *class* and the spec.”

#### Demographics (*n* = 35)

Membership in nondiegetic social groups—those that do not necessarily belong to the gameworld, most clearly *gender* constructions—often reflected in avatar *appearance*. These markers often appeared in relation to backstories, as with “slightly older *looking man* with long gray hair a well as gray facial hair he *appears* rough hardened through years of fighting” and “much more Mercenary in *appearance* and action as opposed to the mindful shopkeep he was, though he still sets up shop on occasion.”

#### Power (*n* = 23)

References to strength and force in terms of *combat* abilities or weapons (“exceptional control and utility in *combat*” or “high *powered* silenced Recon Sniper”), narrative framings of those abilities (“sweetest girl in all the land with unmatched *power* of the tides of healing”), or specific kinds of gear (“Best BBQ and *power* armor in the game.”).

#### Life (*n* = 12)

*Life*, as ascribed to either player (usually “real *life*”), to avatars (“has taken on a *life* of her own”) or referred to in general (“the purpose of *life* is to have fun”).

Somewhat similar to the Symbiote model, the Other model was diffuse—interpreted as reflecting the variability that comes in describing avatars as discrete social others. Interestingly, the most complex and prevalent cluster was features from rich descriptions of avatar appearances, indicating internalization of avatars as cohesively embodied characters. However, most *central* to the model as a whole is the cluster representing avatar backstories, again (as with the Symbiote model) linking a grouping of embodied-description clusters (features, demographics, and power) with more agency-indicative clusters. Notably, this core backstory cluster was tied directly to the cluster indicating authorship, indicating that although the sociality of avatars-as-Other relies on a perception of anthropomorphic autonomy (Banks et al., 2019), the avatar is still internalized as a thing made by the player and even allowed to exist authentically in the PAR through the player’s suspension of disbelief (Banks and Bowman, 2016b).

## Discussion

When we play videogames, we often enter a digital world through an avatar—a representation of our agency in that space, where the relationship between player and avatar is phenomenologically complex. Although much attention has been given to the psychological merging of player and avatar (in which the former comes to identify as the latter), emerging perspective has suggested that players can relate to their avatars in variably social ways. Ranging from asocial Object and merged Me to blended Symbiotes and differentiated Others, these PAR categories have been found across several studies. Less explored, however, is how players understand and internalize avatars—i.e., the subjective interpretation of exactly what they are in a relation *with*. The nature and implications of internalization can be discerned by inferring the mental models that players have for avatars within each of these relational categories. To this end, we engaged a secondary analysis of *N* = 1,201 avatar descriptions, discovering how players in different PAR types internalize elements of avatars. For the balance of this paper, we compare the four PAR types’ inferred MM components, providing critical inferences as to how player-avatar bonds are associated with fundamentally different understandings of what avatars are and why they matter. This discussion is anchored in Table 1, which presents a matrix of aggregate MM clusters across the four PAR types, with semantically similar clusters presented in shared rows and distinctions between these clusters represented in cluster labeling.

**Table 1:** Matrix of mental model clusters across PAR types.

	Object	Me	Symbiote	Other
Demographics	Demographics	Demographics/Gender	Demographics	Demographics
Features	Features/Skin	Features/Face	Features	**Features**
Time	Tenure	**Trajectory**	Trajectory/Time	
Appearance	Aesthetic	Appearance	Aesthetic/Appearance	
Clothing	Armor	Attire	Gear	
Creative Agency	Agency/Crafting	Change		Authorship/Resources
Significance	**Centrality**	Value		
Character Narrative			**Persona**	Backstory
Power			Power	Power
Game Dynamics		Playstyle		Gameplay
Liminality			Liminality/Influence/Role	Life
Gratification	Gratification			

*Labels in bold are the most central (i.e., semantically well connected) concept-clusters for that PAR type*.

### Common Mental Model Clusters Across all PAR Types

Two categories of clusters emerged from avatar descriptions across all PAR types—demographics and features. Notably, although these clusters appeared universally, they did so in different permutations across the four PARs such that knowledge tokens suggest variance in how the clusters formed.

#### Demographics

For all PAR types, players discussed the demographic properties of their avatars. However, there was a meaningful distinction among PAR types in how these demographics were incorporated into mental models as inherent or external to the game’s diegetic frame (Wolf, 1997). Demographic clusters for Object and Me relations were primarily diegetic markers, detailing the races, classes, and levels that help to organize avatars within the gameworld—all as part of more rote descriptions or suggestive of how avatars are situated in the world. Notably, Object relations emphasized avatar sex and race labels while Me relations included the more tightly clustered race, class, and level labels and an entire separate cluster devoted to gender. Thus, although both more asocial PARs relied on diegetic avatar-group markers, demographic clustering differences suggest that for Object relations these markers are more functional identifiers while for Me relations they may be more complex assemblages of (perhaps shared) social-group membership. This aligns with these PARs’ gameplay activities to be mostly focused on either challenge and competition (Object players: Banks, 1995) or immersion and presence (Me players: Banks, 1995; Schuman et al., 2016). In relation to gameplay, Object-PAR demographic labels function much in the same way that one might describe the different pieces on a chessboard—gamepiece materials, such as “ivory” and “ebony,” classify team membership and class labels, such as “knight” or “pawn,” encapsulate function—as allusions to particular ludic roles. In turn, Me relations appear to be marked by demographics as situated in the gameworld (another keyword in that cluster) that are engaged to limit, mark, and frame avatars’ capabilities and role in that world. Acknowledging Me PARs’ tendencies to emphasize identification with avatars (Banks, 1995), these demographics could function as identity assemblages by which players may see themselves in that world.

For Symbiote and Other relations, discussion of demographics trended toward more nondiegetic demographics (i.e., those that may apply to players themselves) and in each case, the demographic cluster was linked to another cluster through a link between gender and appearance keywords. This suggests that, for more social PARs, demographics are important to internalizations of avatars as more socially real (i.e., not merely ludic) personas. This aligns with Symbiote and Other relations’ tendencies to emphasize avatars as partly or wholly independent beings that boast rich identities (Banks, 1995).

#### Features

Feature clusters appeared across all PAR types. They tended to have a greater number of constitutive keywords and to be more tightly networked, compared to other clusters, containing relatively similar contents, such as clothing, face, hair, eyes, height, build, and skin. That said, the relative complexity of these clusters seemed to vary as a function of PAR type, suggesting that descriptions of avatar features were somewhat scaled depending on the degree to of relational sociality.

The more social PARs (Symbiote and Other) exhibited singular feature clusters, where each was a tight-knit collection of many attributes that (as with demographics) suggest consistently rich descriptions of avatars as complex personas, inclusive of their embodied features. This is especially so for Other relations for which the features cluster as the most robust in the model. Interestingly, the more asocial PARs (Object and Me) also exhibited formal feature clusters, but they consisted of fewer attributes and the respective models also exhibited linked secondary feature clusters: a skin cluster for Object and a face cluster for Me. That skin is literally a surface-level characteristic corresponds with Object relations’ tendency to focus more on gameplay and less on the avatar as a persona (reflecting the self or otherwise), while the face as a key differentiator by which people come to know oneself and differentiate among others (Ahn, 2018) reflects Me relations’ marked identification with avatars. Indeed, for those relations, height and eyes are somewhat residual features not captured by other more focused clusters.

### Common Mental Model Clusters Across a Majority of PAR Types

Three mental model clusters were observed in three of the four PAR types: time, appearance, and clothing were present in all but the Other model, while creative agency was present for all but the Symbiote model. Notably, that these three clusters were *not* present for Other players may be reflective of player investment—how time and energy are spent to advance avatars as collections of items that reflect preferences, tastes, and skills.

#### Time

Time-related concept clusters comprised indications of avatars’ tenure as persistent presence in gameplay activities (especially as a project or achievement, for Object relations) or of its trajectory over the course of (often years long) gameplay careers (for Me and Other relations). Notably, the tenure and trajectory clusters for Object and Me relations, respectively, both contain *friends* tokens, indicative of known tendencies for more asocial PARs to emphasize cooperative/competitive and social play (Banks, 1995)—the former using their avatar-as-Object as a play piece to game with others, and the latter using their avatar-as-Me to socially bond with others. While Object relations’ tenure cluster emphasized longstanding use of avatars, Me and Symbiote relations’ clusters included references to *time* or *playtime* that is indicative of players’ felt investment in avatars—so much so that for the Symbiote model it parceled out as a separate cluster. This aligns with Symbiotes’ more social orientation to avatars, such that notions of time being spent together appear to be more salient overall, compared to other PARs. For all, keywords pertaining to time, play, starting, and origins indicate that avatars’ trajectories are semantically entangled with players’ own trajectories within the gameworld. This appears to be especially important for Me relations, as the trajectory cluster was the most robust in the model.

#### Appearance

Clusters conveying internalization of avatars’ holistic visual impression came in two forms: aesthetic and appearance. The former represents an overall *look* that tends to be inherent to a *race* and class (e.g., a dark aesthetic or one common to female blood elves) and the latter more shorthand references to overall look or appreciation thereof (e.g., looking awesome or like oneself). Importantly, these clusters engage avatar appearance heuristically, such that even in the face of complex clusters including myriad tokens for avatar appearance, players still draw on shorthand impressions or tropes to characterize design aims or impressions. These clusters are, however, notably absent in Other relations, where the model for that PAR type instead (as discussed above) included detailed clusters for appearance. So, while most relations engage appearance somewhat heuristically, Other relations (grounded in seeing avatars as authentic social agents) emphasize internalization of appearance details and coordinated features.

#### Clothing

Clusters depicting clothing differed in meaningful ways across these PARs, aligning clearly with core gameplay motivations differentiating the types (Banks, 1995). Object relations focused on *armor* as primarily functional clothing that is less relevant as character or socioemotional marker and more semantically framed as items with ludic value, earned sets, or achievement markers. Said another way, the Object model exhibits that players in this PAR type leverage avatar clothing as a device for creating and controlling a competitive and purpose-built in-game presence. In Me relations, clothing was discussed more as one might discuss a coordinated outfit or ensemble—perhaps just as one would be interested in coordinated representations of oneself (e.g., Goffman, 1959). A deeper read of these narratives and core keywords suggests knowledge tokens much in line with the “proximity of clothing to self” (Sontag and Schlater, 1982)—clothing seen as a component of and external validation of one’s self-concept or self-worth, as well as a symbol of one’s preferences, as there were varied references to liking the outfits, outfitting habits, or aligning outfits with gameplay activities. Finally, for Symbiote relations, discussions of clothing as *gear* (common videogame language describing a variety of “equippable” items, such as clothing, weapons, or accessories) generally leaned toward either functionality or aesthetics, but sometimes engaged both qualities. Such descriptions are not dissimilar from how uniforms or other types of clothing are understood in professional settings (e.g., in a hospital setting, auxiliary workers wearing hospital-issued uniform scrubs while medical students wearing decorated white coats over brand-named clothing), in which clothing can manifest as a normative symbol of professional and sociocultural stratification and distinction (Jenkins, 2014).

#### Creative Agency

The creative clusters for Object and Me players emphasize crafting and personal agency in enacting progress and change in avatars, while Other relations instead focus on drawing from relevant diegetic resources in the *authoring* of avatars. This Other-PAR authoring emphasizes original creation at the intersection of gear, game, and story such that those players’ investments are less about time and activity and more about a commitment to crafting a cohesive persona that exists separately from the player.

### Distinctive Mental Model Clusters Between PAR Types

To this point, we have discussed the resonance of clusters across PARs, with special attention to variance in the constituency of those shared clusters even when the clusters themselves persist across PAR types. However, for the remaining seven of our 12 categories (Table 1), PAR types generated more distinction that they did cohesion. On the surface, such distinction is further evidence that the heuristic PAR types represent qualitatively different approaches to avatars’ role in gameplay—either as a ludic-functional body or the legitimate form of a social other. This distinction is discussed below, along with remarks on clusters that were idiosyncratic to some PAR types.

Object and Me MMs (i.e., those for the more asocial PARs) included clusters emphasizing the significance of avatars in gameplay, where Object relations focused on the functional significance (i.e., the centrality to play, usually as a “main” avatar) and Me relations focused on personal value (as a “favorite”) or legitimacy. Importantly, the concept cluster representing centrality to gameplay was the most robust cluster for the Object model, signaling the importance of having a focal avatar-as-gamepiece to focus on for gameplay (Banks and Bowman, 2015). In contrast, Symbiote and Other models (i.e., those for more social PARs) included clusters emphasizing avatars’ status as characters (personas grounded in backstories, versus gamepieces) and notions of power—both as a personality trait and as a ludic force. Indeed, the persona cluster was the most robust of all clusters in the Symbiote model, signaling the importance of a cohesive character as a bridge as the relation is characterized as being “part of each other.” This stark parceling of clusters reflects the fundamental underpinnings of the PAR framework (Banks, 1995): Asocial relations emphasize the ego as it engages in ludic activities while social relations reflect deeper engagement of the avatar-as-character in narrative activities. Importantly, the game and gameplay are accounted for across the spectrum (as “game” concepts are embedded in other clusters). However, Me and Other models, respectively, illustrate a parceling out of playstyle (affinity and skill in the method of playing) and gameplay (the activities and processes inherent to playing). This supports the PAR-framework assertion that asocial PARs are associated with more egoistic and goal-oriented play and social PARs more with processual and affect-oriented play.

Finally, some clusters were idiosyncratic to PAR types. The Object model was the only to include a cluster clearly depicting gratifications, where the emphasis on enjoyment highlights the hedonic nature of those players’ gaming activities. In contrast, Symbiote and Other models featured clusters representative of avatars’ liminality—their situatedness between the “real” and unreal, having a kind of aliveness, and functioning as a mediator in relations with both game characters and other players.

### Limitations and Future Research

Our findings above should be interpreted with proper consideration of the current work’s limitations. There are, of course, the standard limitations of the method—survey-elicited data are subject to risks around reliability of self-reports and the broad elicitations could have garnered different interpretations of what it means for one to “describe” an avatar. Perhaps, the most pressing limitation is that as a secondary data analysis, we were unable to further probe participants to better understanding their unique gaming experiences. For example, at a descriptive level, we do not have data on participants prior gaming history (either with a focal avatar or with gaming broadly). Likewise, we did not sample purposefully from different videogame genres or videogame properties and thus, we could not control the homogeneity or heterogeneity of gaming experiences (for example, focusing on unique attributes of a given videogame that might influence how players take up and relate to an avatar). With respect to variance in PAR types between videogames, prior research has not found that their frequency varies significantly as a function of game genre (comparing MMORPGs to first-person shooters and other types of role-playing games; Bowman et al., 2016), although this early research did not focus on specific games. Importantly, because we engage data here according to aggregate semantic-network models such that the unit of analysis is the categorical PAR type, we cannot make claims about specific player-avatar relations or other variations within PAR types.

With these limitations in mind, the present findings provide a conceptual and empirical ground for more complex approaches to understanding PARs. Across PAR types, it is broadly clear that what constitutes an avatar is fundamentally different—the avatar-assemblage is internalized differently as a function of player-avatar sociality. Extant literature is rich with discussions of creation and customization as key to avatar engagement, but even those notions differ among PAR types (e.g., creation as crafting vs. authorship, detailed versus heuristic references to appearance). In some cases, notable clusters were either present or absent in PAR-aligned ways—those in Object relations internalized avatar-specific gratifications and Symbiote and Other relations held salient the relative positionality of avatars within narrative worlds. Perhaps even more revealing (and potentially more complicating) are those scenarios in which clusters appeared across all PAR types but reflected differently internalized assemblages. Both demographics and features were broadly relevant across the sample, but for very different reasons: At lower levels of sociality, these clusters indicate mostly descriptive internalizations (for quickly identifying one’s Object or finding oneself among crowds of avatars); at higher levels of sociality, those clusters represent more detailed and specific mental models critical to how players see the avatar as a complex persona. In short, these patterns indicate there is not a consistently monolithic body with which players are connection, but a cadre of diverse avatar-assemblages varying in likely meaningful ways. Future work should explore how the content and structure of these sociotechnical assemblages—in part as well as whole—may reflect antecedents, processes, and effects of gameplay at the individual player level. Regarding players’ abilities to engage in assembly, we could investigate the extent to which avatar customization and creation systems present options that enhance (or perhaps, even hinder) enjoyment and appreciation by making PAR-relevant features more or less available and manipulable.

## Conclusion

From similarities in considering demographics to differences in how armor is discussed, the present data support the four-category typology of player-avatar relations along a continuum of self-differentiated sociality—the similarities and differences in aggregate models for those PARs vary in ways that align with that model. Most importantly, data illustrate that player-avatar relations are grounded in fundamentally different internalizations of what avatars are and why they matter, as varied assemblages of social/technical, material/semiotic, ludic/narrative, and digital/physical components. Because avatars are internalized by players as meaningfully different assemblages, it is critical that they be examined as such. That is, understanding relations with avatar-assemblages requires some bit of scholarly assembly itself, toward understanding the discrete and aggregated contributes of avatar components to play experiences.

## Data Availability Statement

The original contributions presented in this study are included in an online data repository: https://osf.io/8n9mp/. Further inquiries can be directed to the corresponding author.

## Ethics Statement

Ethical review and approval was not required for the study on human participants in accordance with the local legislation and institutional requirements. Written informed consent for participation was not required for this study in accordance with the national legislation and the institutional requirements.

## Author Contributions

JB conducted the Leximancer analyses. JB and NB contributed equally to all other aspects of this manuscript and approved the submitted version.

### Conflict of Interest

The authors declare that the research was conducted in the absence of any commercial or financial relationships that could be construed as a potential conflict of interest.

## Footnotes

^1^As noted in Bowman and Banks (2021), the terms avatar and character are often used interchangeably. That said, *avatar* refers broadly to a digital representation of the player in a gaming environment, while *characters* are a specific kind of avatar whose personality, appearance, and narrative trajectory is crafted by the game (i.e., an existing character). For this manuscript, we use *avatar* in a broad sense, except in specific instances where distinctions are important.

^2^In past work (e.g., Banks and Bowman, 2015), we have characterized the Me relation as “parasocial” in line with Lewis et al. (2008) characterization of psychologically merged relations as parasocial. However, in reflecting on some collegial critiques, we have since abandoned this notion because parasociality requires the relation to be one-way and imaginary, where we characterize PARs as functionally and actually social, where variations come not in whether or not they are dyadic but whether they are perceived as such.
